# Whole blood microRNA expression associated with stroke: Results from the Framingham Heart Study

**DOI:** 10.1371/journal.pone.0219261

**Published:** 2019-08-08

**Authors:** Joel Salinas, Honghuang Lin, Hugo J. Aparico, Tianxiao Huan, Chunyu Liu, Jian Rong, Alexa Beiser, Jayandra J. Himali, Jane E. Freedman, Martin G. Larson, Jonathan Rosand, Hermona Soreq, Daniel Levy, Sudha Seshadri

**Affiliations:** 1 The Framingham Heart Study, Framingham, Massachusetts, United States of America; 2 The Henry and Allison McCance Center for Brain Health, Department of Neurology, Massachusetts General Hospital, Harvard Medical School, Boston, Massachusetts, United States of America; 3 Section of Computational Biomedicine, Department of Medicine, Boston University School of Medicine, Boston, Massachusetts, United States of America; 4 Department of Neurology, Boston University School of Medicine, Boston, Massachusetts, United States of America; 5 Department of Biostatistics, Boston University School of Public Health, Boston, Massachusetts, United States of America; 6 Department of Medicine, University of Massachusetts Medical School, Worcester, Massachusetts, United States of America; 7 Department of Mathematics and Statistics, Boston University, Boston, Massachusetts, United States of America; 8 Center for Genomic Medicine, Massachusetts General Hospital, Boston, Massachusetts, United States of America; 9 Department of Biological Chemistry, The Life Sciences Institute, The Hebrew University of Jerusalem, Jerusalem, Israel; 10 The Edmond and Lily Safra Center for Brain Sciences, The Hebrew University of Jerusalem, Jerusalem, Israel; 11 The Population Sciences Branch, Division of Intramural Research, National Heart, Lung, and Blood Institute, Bethesda, Maryland, United States of America; 12 Glenn Biggs Institute for Alzheimer’s and Neurodegenerative Diseases, University of Texas Health Sciences Center, San Antonio, Texas, United States of America; IRCCS-Policlinico San Donato, ITALY

## Abstract

Emerging evidence suggests microRNAs (miRNAs) may play an important role in explaining variation in stroke risk and recovery in humans, yet there are still few longitudinal studies examining the association between whole blood miRNAs and stroke. Accounting for multiple testing and adjusting for potentially confounding technical and clinical variables, here we show that whole blood miR-574-3p expression was significantly lower in participants with chronic stroke compared to non-cases. To explore the functional relevance of our findings, we analyzed miRNA-mRNA whole blood co-expression, pathway enrichment, and brain tissue gene expression. Results suggest miR-574-3p is involved in neurometabolic and chronic neuronal injury response pathways, including brain gene expression of *DBNDD2* and *ELOVL1*. These results suggest miR-574-3p plays a role in regulating chronic brain and systemic cellular response to stroke and thus may implicate miR-574-3p as a partial mediator of long-term stroke outcomes.

## Introduction

Stroke—ischemic stroke and hemorrhagic stroke—remains a leading cause of mortality and long-term disability worldwide.[1] Treatment efficacy and functional outcome rely heavily on early detection as well as understanding contributors to observed clinical variation in the weeks and years after stroke onset. Biological risk factors leading up to an acute stroke and multiple signaling cascades triggered throughout the brain after stroke influence the balance between neuropathological and neuroprotective processes. However, the majority of these pathways are not yet elucidated.

MicroRNAs (miRNAs) are a class of small (approximately 22 nucleotides), endogenous, non-coding RNA that regulate post-translational gene expression by altering mRNA transcripts.[2] Non-coding RNA networks are critical for molecular responses to disease-related genetic and environmental exposures, and the importance of miRNAs in cerebrovascular disease is emerging. MicroRNAs exist in neurons and other cells of the central nervous system and can be released by these cells into extracellular spaces. Due to pragmatic differences in accessibility, circulating miRNAs may be more clinically relevant than miRNAs derived directly from the central nervous system. MicroRNAs can be detectable in *plasma*, a predominantly extracellular miRNA source, and *whole blood*, a cellular miRNA source with cells like peripheral blood mononuclear cells. Whereas whole blood miRNAs primarily reflect miRNA expression of these cells, whole-blood-derived miRNA expression profiles likely also reflect important interactions between brain pathological processes and systemic factors, such as vascular inflammation or environmental exposures.[3] Much of this work is isolated to animal models and relatively small incipient stroke cohorts, thus the molecular mechanisms underlying many differentially expressed miRNAs remain unclear.[4, 5] Identifying and validating latent biomarkers to study these pathways is ideally undertaken with the use of a large, deeply phenotyped, longitudinal study with participants at risk of developing stroke. In the Framingham Heart Study (FHS), one of the oldest and closely monitored observational community-based cohorts in the United States, several plasma and whole blood miRNAs were associated with age,[6] cancer,[7, 8] cardiovascular disease,[9, 10] and cardiometabolic risk factors.[11] One recent FHS study identified 6 plasma-derived extracellular miRNAs associated with stroke.[12] To clarify the relationship between whole blood miRNAs and cerebrovascular disease, we tested the hypothesis that whole-blood-derived miRNA expression levels would be associated with stroke in a sample of FHS participants.

## Materials and methods

### Study design

FHS is a community-based family study that was initiated in 1948 and has since enrolled three generations of participants.[13, 14] For the present investigation, we focused on the Offspring cohort, who are children of the Original cohort or the spouses of Original cohort children.[13] The Offspring cohort participants enrolled in 1971 and are evaluated about every 4 to 8 years. All participants provided informed consent and the methods were carried out in accordance with the examination protocols approved by the Boston University Medical Center Institutional Review Board.

We examined miRNA cross-sectional and prospective associations with the two most common types of stroke worldwide, ischemic stroke and hemorrhagic stroke; subarachnoid hemorrhage was excluded from the present study given substantially different pathophysiology, age of onset, course of treatment, and outcomes.[15, 16] In FHS, a minority of incident strokes are hemorrhagic stroke, thus stroke cases in our study sample largely represent ischemic stroke.[17] Stroke severity was classified by neurological deficits found on examination during the acute stroke presentation and was further classified into four categories: none (no deficit), mild (deficit in visual, motor, sensory, or language domains, but without functional impairment), moderate (deficit requiring assistance in one domain), and severe (deficit requiring assistance in at least two domains).[18] Full details regarding the FHS stroke surveillance protocol, including diagnosis, classification, and severity assessment were published previously.[17]

Data are available on BioLINCC and access can be requested via the BioLINCC website (https://biolincc.nhlbi.nih.gov/home/). A summary of available data and links to request data for the Framingham Heart Study Offspring Cohort are available at https://biolincc.nhlbi.nih.gov/studies/framoffspring/?q=framingham%20heart%20study. The Religious Orders Study and Rush Memory and Aging Project third-party datasets analyzed during the current study are available in the Accelerating Medicines Partnership for Alzheimer's Disease (AMP-AD) Target Discovery Consortium data portal. These datasets are available upon request and can be accessed at doi:10.7303/syn2580853. Our results are reported in accordance with STROBE guidelines.[19]

### MicroRNA expression profiling

Full details of FHS miRNA expression profiling and quality control have been described previously.[20] In brief, whole blood from fasting morning samples were collected from Offspring participants who attended exam cycle 8 (2005–2008). A total of 346 miRNAs were profiled using quantitative real-time polymerase chain reaction (qRT-PCR) encompassing all commercially available TaqMan chemistry-based miRNA assays in the Gene Expression Core Laboratory at the University of Massachusetts Medical School. QRT-PCR reactions were performed using a high-throughput instrument (BioMark; Fluidigm, San Francisco, CA). The FHS Systems Approach to Biomarker Research in Cardiovascular Disease Initiative Steering Committee reviewed all quality control measures. As in prior experiments showing excellent reproducibility using the BioMark dynamic array platform in conjunction with multiplexed reverse transcriptase reactions for miRNA profiling, we did not encounter cross-contamination with this platform.[9] For replicates, >95% of data points had coefficients of variation <10% (mean ~4%). MicroRNA expression was quantified using cycle threshold (Ct), where higher Ct values represent lower microRNA expression levels. MicroRNAs with a Ct value of 27 or higher were set as missing. A total of 257 miRNAs were expressed in at least 30% of samples and used in the current analysis. We adjusted for batch effects in regression models and did not perform normalization on raw Ct values.

### MicroRNA and mRNA co-expression analysis

Our group analyzed mRNA co-expression in secondary analysis to understand post-translational miRNA gene expression regulation in stroke. The details of mRNA expression profiling have been previously described.[20] The Affymetrix Human Exon 1.0 ST array was used to quantify mRNA expression levels of 17,873 unique transcripts from whole blood RNA using the same participant samples that were collected for miRNA expression profiling. The association between microRNA and mRNA expression was assessed by linear mixed effects modelling adjusted for age, sex, and technical covariates, including isolation batch, RNA concentration, RNA quality, and 260/280 ratio (defined as the ratio of absorbance at 260 and 280nm measured with spectrophotometer). To address the potential for false positive error with multiple comparisons, we used Bonferroni correction to define statistical significance as *P*<2.8x10^-6^ (0.05/17873). The enrichment of biological pathways were performed by WebGestalt,[21] and the KEGG pathways were interrogated.[22]

### Statistical analysis

**Fig 1:**
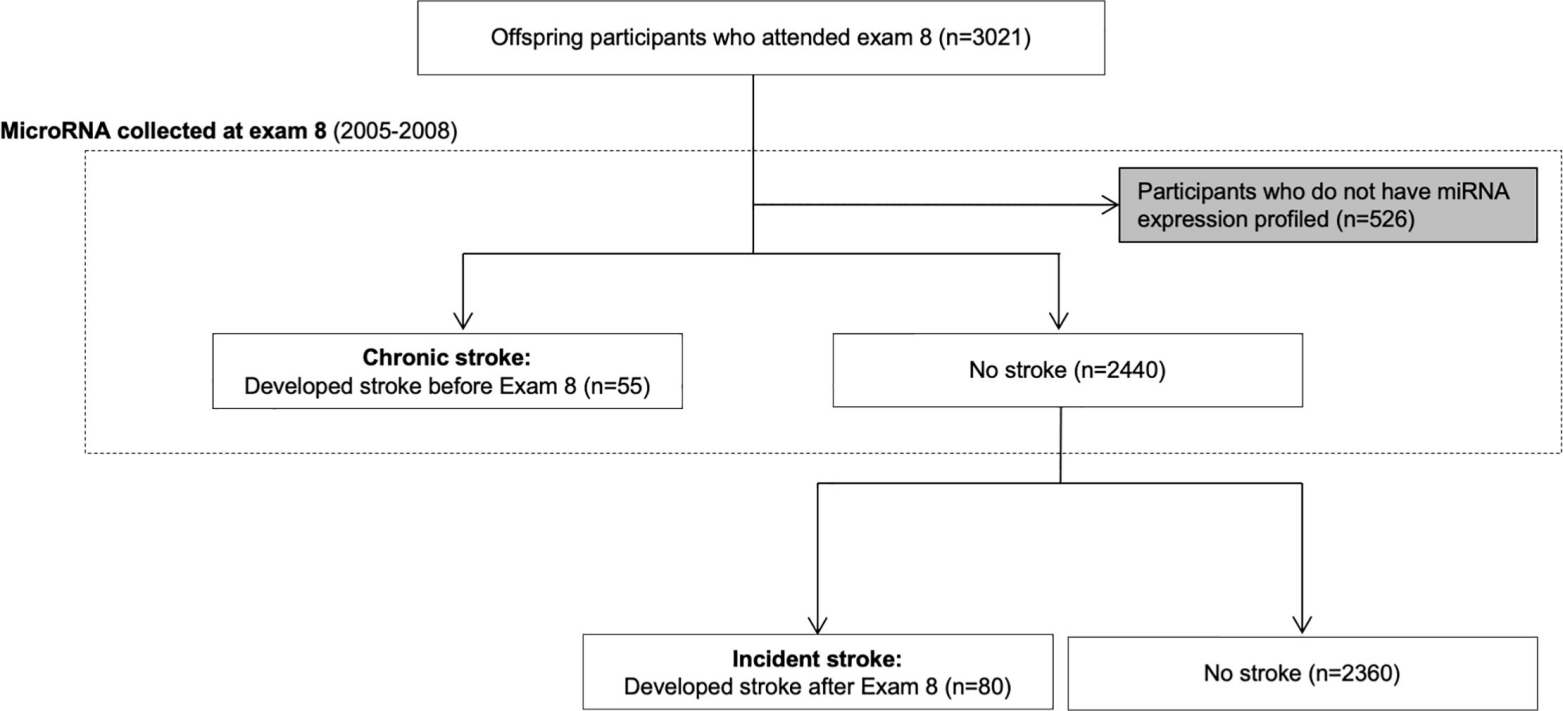
Flow diagram of analytic sample used for analysis.

We separated the analysis between chronic stroke and incident stroke given expected differences in underlying pathophysiologic processes *leading* to an acute stroke and the multiple signaling cascades triggered throughout the brain and systematically *after* stroke. For derivation of analytic samples see Fig 1.

In cross-sectional analysis, chronic stroke was defined as those who had a stroke before RNA collection, which occurred at exam 8. In prospective analysis, incident stroke was defined as those who developed stroke after RNA collection at exam 8. For chronic stroke analyses, because miRNA was collected *after* stroke, we used linear mixed effect models to examine the association of prior stroke with miRNA expression profiles, where stroke status was used as the exposure and each miRNA was used as the dependent variable. Meanwhile for incidence analyses, because miRNA was collected *before* stroke, we used Cox proportional hazards models with robust sandwich estimators for time-to-stroke to examine the prospective association of miRNA expression with future stroke. The miRNA was used as the exposure and time-to-stroke was used as the dependent measure. Censoring factors were death before new-onset stroke and, if no report of stroke, last time of follow-up through December 31^st^, 2013.

Whereas miRNA expression can be dramatically altered in response to stroke, the implications of associated risk factors are often not taken into account.[23] To address this limitation, our study included miRNA profiles of individuals from different age groups with risk factor heterogeneity and adjusted statistical models for age, sex, and potentially confounding clinical variables: systolic blood pressure, hypertension treatment, diabetes, previous cardiovascular disease, atrial fibrillation, and smoking.[24] To account for factors related to RNA processing, we additionally adjusted analyses for isolation batch, RNA quality, concentration, and 260/280 ratio. Using Bonferroni correction, we defined statistical significance as *P*<1.9x10^-4^ (0.05/257 miRNAs) to account for potential false positive error from multiple testing.

## Results

The characteristics of 2495 FHS Offspring participants in the study sample are shown in **Table 1**.

**Table 1:** Clinical characteristics of participants.

Variable	No Stroke (n = 2360)	Chronic Stroke (n = 55)	Incident Stroke (n = 80)
Age, years	66±9	73±9	73±8
Men, n (%)	1051 (45%)	31 (56%)	40 (50%)
Body mass index, kg/m^2^	28±5	28±5	29±5
Systolic blood pressure, mm Hg	128±17	131±19	135±15
Diastolic blood pressure, mm Hg	74±10	68±9	72±10
Current smoking, n (%)	203 (9%)	6 (11%)	2 (3%)
Chronic myocardial infarction, n (%)	110 (5%)	9 (16%)	7 (9%)
Chronic heart failure, n (%)	61 (3%)	5 (9%)	3 (4%)
Chronic diabetes mellitus, n (%)	367 (16%)	23 (42%)	19 (24%)
Antihypertensive treatment, n (%)	1118 (48%)	44 (78%)	51 (65%)
Total cholesterol, mg/dL	186±37	174±40	179±38
Hyperlipidemia treatment, n (%)	1034 (44%)	35 (64%)	43 (54%)
Chronic atrial fibrillation, n (%)	156 (7%)	10 (18%)	14 (18%)
Stroke severity^a^, n (%)			
None	-	14 (25%)	4 (5%)
Mild	-	34 (62%)	42 (53%)
Moderate	-	7 (13%)	9 (11%)
Severe	-	-	25 (31%)

Data are presented as means ± standard deviation or number (percentage).

^a^For stroke severity: none = no deficit; mild = deficit present in visual, motor, sensory, or language domains, but without functional impairment; moderate = deficit requiring assistance in one domain; severe = deficit requiring assistance in at least two domains.

The study sample was 55% women and had mean age 66 years (range from 40 to 92 years). Our cohort included middle-age to older adults with a modest burden of cardiovascular disease risk factors commonly reflected in community-based samples. A total of 55 participants had stroke prior to the RNA collection and, afterwards, an additional 80 participants developed stroke. The mean time from stroke to miRNA collection was 7.8 years (range 0.4 to 34.5 years). The mean time from miRNA collection to incident stroke was 3.2 years (range 0.1 to 7.5 years). Mean follow-up time after miRNA collection at exam 8 was 9.4 years (range 0.1 to 10.8 years). Seven of the 25 participants with severe stroke included in the analysis died within 10 days of their stroke. A total of 526 Offspring participants who attended exam 8 were excluded from the analytic sample because they did not have miRNA profiled (due to no agreement for DNA or RNA research, failure of lab experiment, or other technical factors); of these 526 excluded participants, 23 had prior stroke.

As shown in **Table 2**, one miRNA was significantly associated with chronic stroke among 257 miRNAs: miR-574-3p (*P* = 2.6x10^-6^).

**Table 2:** Most significant whole blood miRNAs in association with chronic stroke in Framingham Offspring participants.

miRNA	Cases (No.)	Non-Cases (No.)	Average expression (Ct)		Association results^a^		
			Cases	Non-Cases	Beta	SE	*P*-value^b^
miR_574_3p	53	2383	12.72	10.51	1.81	0.38	2.6E-06
miR_483_3p	24	793	22.13	23.68	-1.41	0.40	5.0E-04
RNU48_b2	54	2407	4.25	3.24	0.31	0.10	2.3E-03
miR_28_5p	54	2397	14.55	12.13	0.47	0.19	1.1E-02
miR_320b	53	2417	11.30	9.22	0.44	0.18	1.6E-02
RNU48_a2	54	2396	4.32	3.48	0.21	0.09	1.7E-02
U6_snRNA_a1	51	2313	8.92	11.13	-0.57	0.25	2.6E-02
RNU48_b1	54	2405	4.24	3.47	0.21	0.09	2.7E-02
miR_324_3p	54	2386	11.69	10.27	0.39	0.18	2.9E-02
miR_625_3p	47	2358	15.15	15.48	-0.66	0.31	3.1E-02

Abbreviations: Ct, Cycle threshold.

^a^Model fully adjusted for age, sex, technical factors, and potentially confounding clinical variables (systolic blood pressure, hypertension treatment, diabetes, previous CVD, atrial fibrillation, and smoking).

^b^Bonferroni *P-*value cutoff = 0.05/257 miRNAs = 1.9E-4

**Fig 2:**
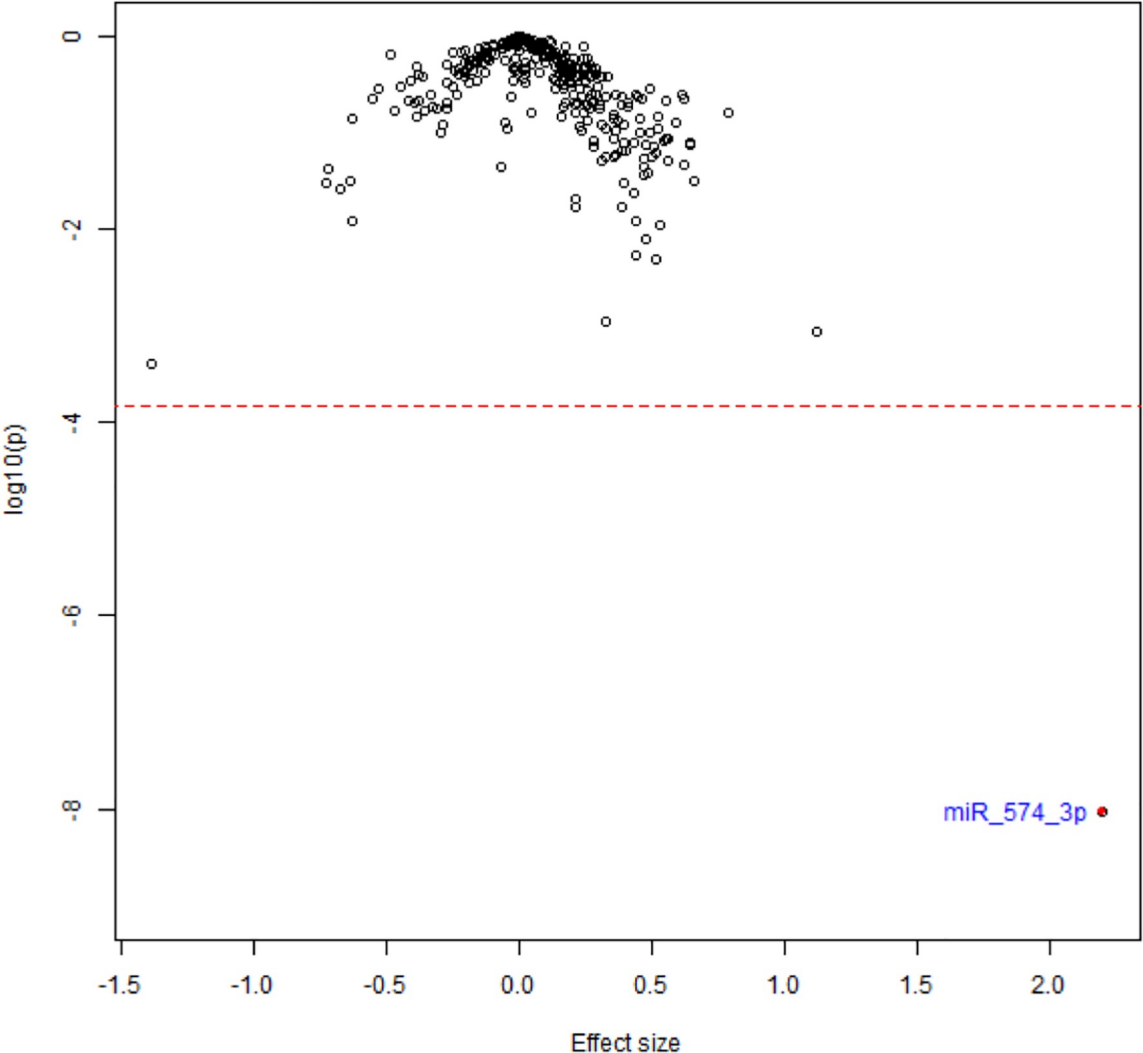
Volcano plot of effect sizes for associations between whole blood miRNA expression profiles and chronic stroke in Framingham Offspring participants.

**Fig 2**illustrates the effect size for this association.

No other miRNAs among the tested transcripts reached the significance cutoff after adjustment for multiple testing (*P*<1.9x10^-4^). **Table 3**shows the top results for incident stroke; no miRNAs showed significant differences after Bonferroni correction, including miR-574-3p (Beta = 0.03, SE = 0.05, *P* = 0.49).

**Table 3:** Most significant whole blood miRNAs in association with incident stroke in Framingham Offspring participants.

miRNA	Cases (No.)	Non-Cases (No.)	Average expression (Ct)		Association results^a^		
			Cases	Non-Cases	Beta	SE	*P*-value^b^
miR_484	80	2323	4.58	4.78	-0.58	0.21	6.4E-03
miR_29b_2_5p	78	2249	21.67	21.02	0.22	0.08	9.3E-03
miR_193b_3p	76	2308	18.23	18.90	-0.18	0.07	1.4E-02
miR_26b_5p	78	2274	18.14	16.76	0.11	0.05	1.9E-02
miR_1271_5p_b1	74	2258	18.40	18.68	-0.18	0.08	3.7E-02
let_7d_3p	37	891	21.11	22.34	-0.16	0.08	4.1E-02
miR_129_1_3p	55	1683	22.93	23.51	-0.18	0.09	4.2E-02
miR_629_3p	73	2286	18.59	19.05	-0.17	0.09	4.7E-02
miR_16_1_3p	63	1765	22.69	22.23	0.14	0.07	4.8E-02
miR_320b	80	2346	9.41	9.21	-0.20	0.10	4.9E-02

Abbreviations: Ct, Cycle threshold.

^a^Model fully adjusted for age, sex, and technical factors, and potentially confounding clinical variables (systolic blood pressure, hypertension treatment, diabetes, previous CVD, atrial fibrillation, and smoking).

^b^Bonferroni *P*-value cutoff = 0.05/257 miRNAs = 1.9E-4

In our secondary analyses, we excluded participants with hemorrhagic stroke (n = 1) to assess potential confounding effects and the result was the same (*P* = 5.8x10^-6^ for miR-574-3p). Our findings remained similar when accounting for the potential confounding effects of medications commonly used after stroke (aspirin, warfarin, and atorvastatin),[25] and miRNA expression profile variance over time after acute stroke by additionally adjusting for the time interval from stroke to miRNA collection; the time interval used for non-cases was 0 years (S1 Table). To assess the potential inverse effects between miRNAs and stroke, we tested the association by treating miRNA expression as the exposure and stroke as the dependent variable. MiR-574-3p remained the most significant miRNA associated with chronic stroke (*P* = 1.0x10^-4^, see S2 Table).

To identify potential targets of miR-574-3p, our group tested the association of miR-574-3p expression with gene expression in whole blood. A total of 1063 genes were significantly associated with miR-574-3p (*P*<2.8x10^-6^). The top 20 genes are shown in S3 Table. To investigate the potential function of these 1063 genes, we identified the top 10 pathways enriched with these genes as shown in S4 Table.

We also examined the enrichment of miR-574-3p-associated genes among predicted miR-574-3p targets. The predicted targets were downloaded from the PITA database.[26] Thirty-four genes were predicted as targets of miR-574-3p. Among them, six were significantly associated with miR-574-3p in whole blood, including *ATPIF1*, *CLTC*, *FOSL2*, *KIAA1033*, *RXRA*, and *SRF*, representing a 2.7-fold enrichment of predicted targets (Fisher’s exact test *P* = 0.03).

Given that different tissues could have different gene expression patterns, we studied the association of miR-574-3p expression with gene expression in brain tissue using Religious Orders Study and Rush Memory and Aging Project data.[27, 28] As shown in S5 Table, the expression of *DBNDD2* and *ELOVL1* in brain tissue was significantly associated with miR-574-3p expression. *ELOVL1* also showed significant association with miR-574-3p in whole blood.

## Discussion

Our study examined the association of 257 whole-blood-derived miRNAs with stroke in a community-based sample of men and women. Adjusting for age, sex, technical factors, and potentially confounding clinical variables, we identified one miRNA—miR-574-3p—for which expression was downregulated in individuals who experienced a stroke before miRNA sampling. The observed association suggests miR-574-3p may play a role in regulating long-term brain and systemic cellular pathophysiologic changes triggered after stroke. Whole blood miRNAs may also be implicated in stroke pathogenesis, though we did not observe significant associations between the whole blood miRNA transcripts tested and future stroke, including miR-574-3p.

Among previous studies of smaller incipient acute-stroke cohorts, circulating whole-blood-derived miR-574-3p was also reported as downregulated in the setting of acute stroke.[29] Our results agree with Sepramaniam et al.’s findings[29] and add to the extant research by further supporting the role of miR-574-3p as an important gene regulator in cerebrovascular disease.[12, 30]

Circulating miRNAs are released by cardiac and endothelial cells in stress states and have been associated with cardiovascular disease, suggesting that functional specificity is responsible for the cellular expression of unique miRNAs.[6, 9–11] In whole blood, cardiovascular disease has been linked to miRNA transcriptional patterns (e.g., co-expression pattern disruption for B-cell-centered immune function in cases of coronary heart disease).[31] As reviewed by Small et al.,[32] various cardiovascular disease settings have been associated with specific changes in miRNA profiles, including myocardial remodeling which typically involves cardiomyocyte hypertrophy (miR-1, miR-21, miR-23, miR-133, miR-208a), cardiomyocyte apoptosis and regeneration (miR-195, miR-199a, miR-320), aberrant cardiac conduction (miR-1), interstitial fibrosis (miR-21, miR29, miR-133a), restenosis (miR-21, miR-145, miR-221), and angiogenesis (miR-221, miR-222, miR-210, miR-126, miR-17-92). More recently, McManus et al. showed significant associations between cardiometabolic risk factor clustering and whole-blood-derived levels of miR-197-3p, miR-328, miR-505-5p, and miR-145-5p.[11] Whereas whole blood miR-574-3p has been associated with promoting vascular smooth muscle cell growth in the progression of coronary artery disease,[33] as expected, whole blood miR-574-3p in our sample was significantly associated with prevalent cardiovascular disease (*P* = 1.4x10^-5^) in post-hoc analysis. Prevalent cardiovascular disease was defined as reported presence or history of diseases related to atherosclerosis at the time of blood collection for miRNA profiling, which includes coronary heart disease, myocardial infarction, coronary insufficiency, intermittent claudication, and congestive heart failure. In a prior FHS study, plasma miR-574-3p was unassociated with cardiovascular disease (i.e., coronary heart disease).[12] In examining other diseases, plasma miR-574-3p levels have been associated with various forms of cancer.[34, 35] However, the time course and extent that whole blood miRNA expression profiles reflect cerebrovascular events remain unclear. MicroRNAs can regulate genes central in thrombogenicity and neuroinflammation, including some implicated directly in stroke pathogenesis.[23, 36] In cell models, miR-574-3p downregulation was associated with remnant-like lipoprotein acceleration of endothelial progenitor cell senescence,[37] though miR-574-3p regulation of identified target genes has yet to be studied in cell models.

Human and animal models suggest miRNA expression profiles can vary by acute phase or recovery phase of stroke, and the miRNA expression profiles that can be used to distinguish between acute or recovery phase typically return to baseline levels over time.[29] In contrast to prior work, we did not observe a significant association between other whole blood miRNAs related with acute stroke.[29, 38–40] Beyond technical and context-dependent differences, disagreement between our findings and other studies may be due to differences between study populations (e.g., Sepramaniam et al. included individuals primarily of East Asian descent).[29] We also did not observe significant associations with the 6 plasma-derived extracellular miRNAs linked with stroke prevalence or incidence.[12] Mick et al.’s study of plasma-derived extracellular RNAs used the same FHS Offspring cohort and found that chronic stroke was associated with 3 miRNAs (miR-877-5p, miR-124-3p, and miR-320d) and 1 small nucleolar RNA (SNO1402), while incident stroke was associated with 3 independent miRNAs (miR-656-3p, miR-3615, and miR-941). Our results complement this literature by examining whole-blood-derived miRNA transcripts associated with stroke. Observed differences between plasma and whole blood miRNA expression profiles associated with chronic stroke are consistent with Shah et al.’s general observation of within-individual discordance between plasma versus whole-blood-derived miRNA transcripts.[30]

Regarding the potential function of the 1063 genes expressed in blood associated with miR-574-3p, many significant genes were enriched in pathways involved in non-alcoholic fatty liver disease (*P* = 2.7x10^-9^) and Alzheimer's disease (AD, *P* = 2.2x10^-7^), suggesting that miR-574-3p might exert its effect through brain-related pathways, particularly neurometabolic function and chronic response to neuronal injury given the association of miR-574-3p expression with *DBNDD2* and *ELOVL1* gene expression in brain tissue. Aside from established links between cerebrovascular disease and increased AD risk,[41] previous investigation has identified associations between atherosclerotic cardiovascular disease outcomes (including stroke) and non-alcoholic fatty liver disease.[42] Potential mechanisms for this association include proatherogenic factors in hepatic steatosis, such as diabetes, hypertension, and dyslipidemia or unmeasured signaling factors released by the liver.[43] Another possible mechanism includes increased inflammation in the setting of hepatic steatosis, which may be involved in early atherogenesis.[44] Visceral adiposity and lipid metabolism and synthesis may also play a role. For example, common low-density lipoprotein receptor polymorphisms have been tied to RNA splicing efficiency in human liver, brain, and AD.[45] Among the two genes expressed in brain tissue associated with miR-574-3p, *ELOVL1* (ELOVL fatty acid elongase 1) regulates very long chain fatty acid synthesis, particularly in the brain, and inhibiting *ELOVL1* with agents like fibrates reduces very long chain fatty acid accumulation in fibroblasts.[46] The second associated gene expressed in brain tissue was *DBNDD2* (dysbindin domain containing 2). *DBNDD2* encodes dysbindin, which is a protein that functions in parallel with the ubiquitin modification system to modulate lysosome biosynthesis and protein turnover and trafficking.[47, 48] *DBNDD2* is implicated in neurodegeneration and neuronal injury as an apoptosis response gene.[47] For example, dysbindin and dysbindin homologues like casein kinase-1 binding protein (CK1BP) are found in ubiquitinated lesions, including neurofibrillary tangles and granulovacuolar degeneration bodies in AD.[48] Supporting this functional relationship, in the present study miR-574-3p was associated with whole blood enrichment of genes more generally involved in response to genotoxic stress, such as cell hypoxia, normal vasculogenesis, and cell cycle regulation. Relevant cellular functions in genotoxic stress response included platelet activation, focal adhesion, regulation of actin cytoskeleton, B cell receptor signaling pathways, and leukocyte transendothelial migration, which are implicated in molecular models of chronic cerebrovascular disease and signaling cascades after brain ischemia.[49]

Whole blood miRNAs may regulate neuronal or glial gene expression directly or indirectly by regulating gene expression in other tissues relevant to stroke, such as peripheral mononuclear cells. In the context of chronic stroke, our findings likely represent a circulating pool of miRNAs determined by downregulated intracellular concentrations of miR-574-3p in peripheral mononuclear cells. Beyond regulating gene expression through translation inhibition, our findings may be explained by increased post-transcriptional degradation of miR-574-3p. Moreover, observed functional associations could be attributed to cell-specific regulation from chronic stroke-related physiological disturbances outside of the central nervous system, such as hepatocytes.

Our results did not support an association between whole-blood-derived miRNA expression profiles and incident stroke, though this does not preclude the existence of a link between the two.[36] For example, single nucleotide polymorphisms (SNPs) associated with stroke may exist outside of intronic gene regions and thus may not be related to gene expression. Furthermore, the limited number of new-onset stroke cases in our sample may explain why our group did not observe a significant association between miR-574-3p and incident stroke. Other explanations for our incidence analysis results include bidirectional relationships between microRNA expression levels and stroke, intermediate mechanisms, differential use of medications affecting miR-574-3p expression, and variations in stroke-related neuroinflammation processes due to inherited or acquired changes in the cholinergic blockade of inflammation (e.g., via common SNPs).[50, 51]

While our findings suggest miR-574-3p may be a mediator of neurometabolic and cytotoxic stress responses to stroke, an alternative explanation for the observed associations is that miR-574-3p may be involved in stroke pathogenesis. However, this explanation is less likely given the present incidence analyses did not show a significant prospective association. A second possibility is that miR-574-3p may be associated with survival; though, in post-hoc analysis miR-574-3p was not associated with survival after stroke in our sample (*P* = 0.65). In testing this association, the most significant miRNA for survival after stroke was miR-18a-5p-a1, but it did not cross the Bonferroni significance threshold either. A third alternative for the observed miR-574-3p association with chronic stroke is differences in stroke severity which would have likely attenuated observed effects. Although participants with milder chronic strokes are more likely to follow-up in cohort studies, our sample had minimal loss to follow-up regardless of stroke severity. Other possible explanations for the observed relationship with chronic stroke are that miR-574-3p is associated with residual confounding from a stroke intervention, acquired or inherited individual biological differences, or other unknown mechanisms not included in our models.

The molecular underpinnings of cerebrovascular disease are complex, because heterotypic SNP-miRNA-mRNA associations are disrupted by brain pathology and broader extrinsic risk factors.[3] Although miR-574-3p was not associated with incident stroke, given prior work suggesting miR-574-3p is a partial regulator in acute stroke and our observed association with chronic stroke, miR-574-3p merits further exploration as a potential biomarker or treatment target for long-term outcomes of cerebrovascular disease. Thus, larger longitudinal samples of participants from diverse backgrounds at risk for new-onset stroke are needed.

Our study has limitations. First, FHS participants are predominantly middle-age adults of European ancestry so the generalizability of our findings, especially to other races and ethnicities or younger individuals, remains unclear. Second, given that whole blood may include miRNA-loaded exosomes and is comprised of different cell types, the miRNA contribution of each cell type is relevant. Our analyses were not adjusted for cell composition because whole blood cell composition was not determined at the time miRNA was quantified. Third, low abundance miRNAs were excluded when not present in at least 30% of samples. Finally, our cross-sectional analysis cannot establish causality or directionality between miRNAs and stroke.

## Conclusions

MiR-574-3p was associated with stroke in a community-based sample of more than 2000 participants. The functional role and regulatory molecular mechanisms of miRNA before, during, and after stroke are not well understood yet investigating these mechanisms may clarify the pathogenic or neuroprotective properties of miRNAs, inform better treatments for cerebrovascular disease, and facilitate better measurement of neuropathology where direct access to tissue for molecular analyses is desirable but not feasible. Additional studies are needed to further verify our findings and identify other miRNAs that may serve as accessible diagnostic or therapeutic targets for cerebrovascular disease.

## Supporting information

S1 Table Associations between whole blood miRNA expression profiles and chronic stroke with additional adjustment for potentially confounding medication use and the time interval between chronic stroke and RNA extraction. Abbreviations: Ct, Cycle threshold.^a^Model adjusted for age, sex, technical factors, time interval between prior stroke and RNA extraction (time interval for non-cases is zero years), potentially confounding clinical variables (systolic blood pressure, hypertension treatment, diabetes, previous cardiovascular disease, atrial fibrillation, and smoking), and common medication use after stroke (aspirin, warfarin, and atorvastatin). ^b^Bonferroni *p* value cutoff = 0.05/257 miRNAs = 1.9E-4.
(DOCX)

S2 Table Most significant whole blood miRNAs in association with chronic stroke by treating miRNA expression as exposure and stroke as the dependent variable. Abbreviations: Ct, Cycle threshold. ^a^Model adjusted for age, sex, technical factors, time interval between prior stroke and RNA extraction (time interval for non-cases is zero years), potentially confounding clinical variables (systolic blood pressure, hypertension treatment, diabetes, previous cardiovascular disease, atrial fibrillation, and smoking), and common medication use after stroke (aspirin, warfarin, and atorvastatin). ^b^Bonferroni *p* value cutoff = 0.05/257 miRNAs = 1.9E-4.
(DOCX)

S3 Table Top genes whose expression in blood was associated with miR-574-3p. A total of 1063 genes were identified (*P*<2.8E-6).
(DOCX)

S4 Table Enrichment of genes expressed in blood associated with miR-574-3p. (DOCX)

S5 Table Top genes whose expression was associated with miR-574-3p in brain samples collected from the ROS/MAP project. Two genes were significant after Bonferroni correction (*P*<8.9E-7).
(DOCX)

## References

[1] Benjamin EJ, Blaha MJ, Chiuve SE, Cushman M, Das SR, Deo R, et al Heart Disease and Stroke Statistics-2017 Update: A Report From the American Heart Association. Circulation. 2017;135(10):e146–e603. 10.1161/CIR.0000000000000485 28122885 PMC5408160

[2] Huan T, Rong J, Tanriverdi K, Meng Q, Bhattacharya A, McManus DD, et al Dissecting the roles of microRNAs in coronary heart disease via integrative genomic analyses. Arterioscler Thromb Vasc Biol. 2015;35(4):1011–21. 10.1161/ATVBAHA.114.305176 25657313 PMC4376567

[3] Rao P, Benito E, Fischer A. MicroRNAs as biomarkers for CNS disease. Front Mol Neurosci. 2013;6:39 10.3389/fnmol.2013.00039 24324397 PMC3840814

[4] Rink C, Khanna S. MicroRNA in ischemic stroke etiology and pathology. Physiol Genomics. 2011;43(10):521–8. 10.1152/physiolgenomics.00158.2010 20841499 PMC3110894

[5] Liu DZ, Tian Y, Ander BP, Xu H, Stamova BS, Zhan X, et al Brain and blood microRNA expression profiling of ischemic stroke, intracerebral hemorrhage, and kainate seizures. J Cereb Blood Flow Metab. 2010;30(1):92–101. 10.1038/jcbfm.2009.186 19724284 PMC2949089

[6] Freedman JE, Ercan B, Morin KM, Liu CT, Tamer L, Ayaz L, et al The distribution of circulating microRNA and their relation to coronary disease. F1000Res. 2012;1:50 10.12688/f1000research.1-50.v1 24358814 PMC3752638

[7] Calin GA, Croce CM. MicroRNA-cancer connection: the beginning of a new tale. Cancer Res. 2006;66(15):7390–4. 10.1158/0008-5472.CAN-06-0800 . 16885332

[8] Farazi TA, Hoell JI, Morozov P, Tuschl T. MicroRNAs in human cancer. Advances in experimental medicine and biology. 2013;774:1–20. 10.1007/978-94-007-5590-1_1 23377965 PMC3704221

[9] McManus DD, Lin H, Tanriverdi K, Quercio M, Yin X, Larson MG, et al Relations between circulating microRNAs and atrial fibrillation: data from the Framingham Offspring Study. Heart rhythm: the official journal of the Heart Rhythm Society. 2014;11(4):663–9. Epub 2014/01/22. 10.1016/j.hrthm.2014.01.018 24444445 PMC4219255

[10] McManus DD, Tanriverdi K, Lin H, Esa N, Kinno M, Mandapati D, et al Plasma microRNAs are associated with atrial fibrillation and change after catheter ablation (the miRhythm study). Heart rhythm: the official journal of the Heart Rhythm Society. 2015;12(1):3–10. Epub 2014/09/27. 10.1016/j.hrthm.2014.09.050 25257092 PMC4277933

[11] McManus DD, Rong J, Huan T, Lacey S, Tanriverdi K, Munson PJ, et al Messenger RNA and MicroRNA transcriptomic signatures of cardiometabolic risk factors. BMC Genomics. 2017;18(1):139 10.1186/s12864-017-3533-9 28178938 PMC5299677

[12] Mick E, Shah R, Tanriverdi K, Murthy V, Gerstein M, Rozowsky J, et al Stroke and Circulating Extracellular RNAs. Stroke. 2017;48(4):828–34. 10.1161/STROKEAHA.116.015140 28289238 PMC5373984

[13] Kannel WB, Feinleib M, McNamara PM, Garrison RJ, Castelli WP. An investigation of coronary heart disease in families. The Framingham offspring study. Am J Epidemiol. 1979;110(3):281–90. 10.1093/oxfordjournals.aje.a112813 . 474565

[14] Wolf PA, Kannel WB, McNamara PM. Occult impaired cardiac function, congestive heart failure, and risk of thrombotic stroke: the Framingham Study. Neurology. 1970;20(4):373 . 5534972

[15] Sudlow CL, Warlow CP. Comparable studies of the incidence of stroke and its pathological types: results from an international collaboration. International Stroke Incidence Collaboration. Stroke. 1997;28(3):491–9. 10.1161/01.str.28.3.491 . 9056601

[16] Krishnamurthi RV, Feigin VL, Forouzanfar MH, Mensah GA, Connor M, Bennett DA, et al Global and regional burden of first-ever ischaemic and haemorrhagic stroke during 1990–2010: findings from the Global Burden of Disease Study 2010. Lancet Glob Health. 2013;1(5):e259–81. 10.1016/S2214-109X(13)70089-5 25104492 PMC4181351

[17] Carandang R, Seshadri S, Beiser A, Kelly-Hayes M, Kase CS, Kannel WB, et al Trends in incidence, lifetime risk, severity, and 30-day mortality of stroke over the past 50 years. JAMA. 2006;296(24):2939–46. 10.1001/jama.296.24.2939 . 17190894

[18] Lin HJ, Wolf PA, Kelly-Hayes M, Beiser AS, Kase CS, Benjamin EJ, et al Stroke severity in atrial fibrillation. The Framingham Study. Stroke. 1996;27(10):1760–4. PubMed 10.1161/01.str.27.10.1760 . 8841325

[19] von Elm E, Altman DG, Egger M, Pocock SJ, Gøtzsche PC, Vandenbroucke JP, et al The Strengthening the Reporting of Observational Studies in Epidemiology (STROBE) statement: guidelines for reporting observational studies. Ann Intern Med. 2007;147(8):573–7. PubMed 10.7326/0003-4819-147-8-200710160-00010 . 17938396

[20] Freedman JE, Gerstein M, Mick E, Rozowsky J, Levy D, Kitchen R, et al Diverse human extracellular RNAs are widely detected in human plasma. Nat Commun. 2016;7:11106 Epub 2016/04/27. 10.1038/ncomms11106 27112789 PMC4853467

[21] Wang J, Duncan D, Shi Z, Zhang B. WEB-based GEne SeT AnaLysis Toolkit (WebGestalt): update 2013. Nucleic Acids Res. 2013;41(Web Server issue):W77–83. 10.1093/nar/gkt439 23703215 PMC3692109

[22] Kanehisa M, Goto S. KEGG: kyoto encyclopedia of genes and genomes. Nucleic Acids Res. 2000;28(1):27–30. PubMed 10.1093/nar/28.1.27 10592173 PMC102409

[23] Li WA, Efendizade A, Ding Y. The role of microRNA in neuronal inflammation and survival in the post ischemic brain: a review. Neurol Res. 2017:1–9. 10.1080/01616412.2017.1327505 . 28552032

[24] Dufouil C, Beiser A, McLure LA, Wolf PA, Tzourio C, Howard VJ, et al Revised Framingham Stroke Risk Profile to Reflect Temporal Trends. Circulation. 2017;135(12):1145–59. 10.1161/CIRCULATIONAHA.115.021275 28159800 PMC5504355

[25] Kernan WN, Ovbiagele B, Black HR, Bravata DM, Chimowitz MI, Ezekowitz MD, et al Guidelines for the prevention of stroke in patients with stroke and transient ischemic attack: a guideline for healthcare professionals from the American Heart Association/American Stroke Association. Stroke. 2014;45(7):2160–236. 10.1161/STR.0000000000000024 . 24788967

[26] Kertesz M, Iovino N, Unnerstall U, Gaul U, Segal E. The role of site accessibility in microRNA target recognition. Nat Genet. 2007;39(10):1278–84. Epub 2007/09/26. 10.1038/ng2135 . 17893677

[27] Bennett DA, Schneider JA, Arvanitakis Z, Wilson RS. Overview and findings from the religious orders study. Curr Alzheimer Res. 2012;9(6):628–45. 22471860 10.2174/156720512801322573 PMC3409291

[28] Bennett DA, Schneider JA, Buchman AS, Barnes LL, Boyle PA, Wilson RS. Overview and findings from the rush Memory and Aging Project. Curr Alzheimer Res. 2012;9(6):646–63. 22471867 10.2174/156720512801322663 PMC3439198

[29] Sepramaniam S, Tan JR, Tan KS, DeSilva DA, Tavintharan S, Woon FP, et al Circulating microRNAs as biomarkers of acute stroke. Int J Mol Sci. 2014;15(1):1418–32. 10.3390/ijms15011418 24447930 PMC3907877

[30] Shah R, Tanriverdi K, Levy D, Larson M, Gerstein M, Mick E, et al Discordant Expression of Circulating microRNA from Cellular and Extracellular Sources. PLoS One. 2016;11(4):e0153691 Epub 2016/04/29. 10.1371/journal.pone.0153691 27123852 PMC4849639

[31] Huan T, Zhang B, Wang Z, Joehanes R, Zhu J, Johnson AD, et al A systems biology framework identifies molecular underpinnings of coronary heart disease. Arterioscler Thromb Vasc Biol. 2013;33(6):1427–34. 10.1161/ATVBAHA.112.300112 23539213 PMC3752786

[32] Small EM, Frost RJ, Olson EN. MicroRNAs add a new dimension to cardiovascular disease. Circulation. 2010;121(8):1022–32. Epub 2010/03/03. 10.1161/CIRCULATIONAHA.109.889048 20194875 PMC2847432

[33] Lai Z, Lin P, Weng X, Su J, Chen Y, He Y, et al MicroRNA-574-5p promotes cell growth of vascular smooth muscle cells in the progression of coronary artery disease. Biomed Pharmacother. 2018;97:162–7. Epub 2017/11/02. 10.1016/j.biopha.2017.10.062 . 29091861

[34] Bryant RJ, Pawlowski T, Catto JW, Marsden G, Vessella RL, Rhees B, et al Changes in circulating microRNA levels associated with prostate cancer. Br J Cancer. 2012;106(4):768–74. Epub 2012/01/14. 10.1038/bjc.2011.595 22240788 PMC3322952

[35] Summerer I, Unger K, Braselmann H, Schuettrumpf L, Maihoefer C, Baumeister P, et al Circulating microRNAs as prognostic therapy biomarkers in head and neck cancer patients. Br J Cancer. 2015;113(1):76–82. Epub 2015/06/10. 10.1038/bjc.2015.111 26057452 PMC4647540

[36] Volny O, Kasickova L, Coufalova D, Cimflova P, Novak J. microRNAs in Cerebrovascular Disease. Advances in experimental medicine and biology. 2015;888:155–95. Epub 2015/12/15. 10.1007/978-3-319-22671-2_9 . 26663183

[37] Yang DG, Liu L, Zhou SH. MicroRNA alterations in senescent endothelial progenitor cells induced by remnant-like lipoproteins. Chin Med J (Engl). 2012;125(19):3479–84. Epub 2012/10/10. . 23044309

[38] Li WY, Jin J, Chen J, Guo Y, Tang J, Tan S. Circulating microRNAs as potential non-invasive biomarkers for the early detection of hypertension-related stroke. Journal of human hypertension. 2014;28(5):288–91. Epub 2013/10/18. 10.1038/jhh.2013.94 . 24132136

[39] Li P, Teng F, Gao F, Zhang M, Wu J, Zhang C. Identification of circulating microRNAs as potential biomarkers for detecting acute ischemic stroke. Cell Mol Neurobiol. 2015;35(3):433–47. 10.1007/s10571-014-0139-5 . 25410304 PMC11486203

[40] Wang W, Sun G, Zhang L, Shi L, Zeng Y. Circulating microRNAs as novel potential biomarkers for early diagnosis of acute stroke in humans. J Stroke Cerebrovasc Dis. 2014;23(10):2607–13. 10.1016/j.jstrokecerebrovasdis.2014.06.002 . 25287657

[41] Zhou J, Yu JT, Wang HF, Meng XF, Tan CC, Wang J, et al Association between stroke and Alzheimer's disease: systematic review and meta-analysis. J Alzheimers Dis. 2015;43(2):479–89. 10.3233/JAD-140666 . 25096624

[42] Mellinger JL, Pencina KM, Massaro JM, Hoffmann U, Seshadri S, Fox CS, et al Hepatic steatosis and cardiovascular disease outcomes: An analysis of the Framingham Heart Study. J Hepatol. 2015;63(2):470–6. 10.1016/j.jhep.2015.02.045 25776891 PMC5282653

[43] Speliotes EK, Yerges-Armstrong LM, Wu J, Hernaez R, Kim LJ, Palmer CD, et al Genome-wide association analysis identifies variants associated with nonalcoholic fatty liver disease that have distinct effects on metabolic traits. PLoS Genet. 2011;7(3):e1001324 10.1371/journal.pgen.1001324 21423719 PMC3053321

[44] McKimmie RL, Daniel KR, Carr JJ, Bowden DW, Freedman BI, Register TC, et al Hepatic steatosis and subclinical cardiovascular disease in a cohort enriched for type 2 diabetes: the Diabetes Heart Study. Am J Gastroenterol. 2008;103(12):3029–35. 10.1111/j.1572-0241.2008.02188.x 18853970 PMC3638961

[45] Zou F, Gopalraj RK, Lok J, Zhu H, Ling IF, Simpson JF, et al Sex-dependent association of a common low-density lipoprotein receptor polymorphism with RNA splicing efficiency in the brain and Alzheimer's disease. Hum Mol Genet. 2008;17(7):929–35. 10.1093/hmg/ddm365 18065781 PMC2361153

[46] Schackmann MJ, Ofman R, Dijkstra IM, Wanders RJ, Kemp S. Enzymatic characterization of ELOVL1, a key enzyme in very long-chain fatty acid synthesis. Biochim Biophys Acta. 2015;1851(2):231–7. 10.1016/j.bbalip.2014.12.005 . 25499606

[47] Lucas T, Pratscher B, Fink D, Wolschek M, Samorapoompichit P, Schofer C, et al The human orthologue of a novel apoptosis response gene induced during rat myelomonocytic stem cell apoptosis maps to 20q13.12. Stem Cells Dev. 2005;14(5):556–63. 10.1089/scd.2005.14.556 . 16305340

[48] Yin H, Laguna KA, Li G, Kuret J. Dysbindin structural homologue CK1BP is an isoform-selective binding partner of human casein kinase-1. Biochemistry. 2006;45(16):5297–308. 10.1021/bi052354e . 16618118

[49] Durukan A, Tatlisumak T. Acute ischemic stroke: overview of major experimental rodent models, pathophysiology, and therapy of focal cerebral ischemia. Pharmacol Biochem Behav. 2007;87(1):179–97. 10.1016/j.pbb.2007.04.015 . 17521716

[50] Hanin G, Shenhar-Tsarfaty S, Yayon N, Yau YH, Bennett ER, Sklan EH, et al Competing targets of microRNA-608 affect anxiety and hypertension. Hum Mol Genet. 2014;23(17):4569–80. 10.1093/hmg/ddu170 24722204 PMC4119407

[51] Soreq H. Checks and balances on cholinergic signaling in brain and body function. Trends Neurosci. 2015;38(7):448–58. 10.1016/j.tins.2015.05.007 . 26100140

