# Supplementary material for: Whole blood microRNA expression associated with stroke: Results from the Framingham Heart Study
Source: PLoS One. 2019 Aug 8;14(8):e0219261. doi: 10.1371/journal.pone.0219261 (PMC6687152; doi:10.1371/journal.pone.0219261)
Supplement: S2 Table — Abbreviations: Ct, Cycle threshold. aModel adjusted for age, sex, technical factors, time interval between prior stroke and RNA extraction (time interval for non-cases is zero years), potentially confounding clinical variables (systolic blood pressure, hypertension treatment, diabetes, previous cardiovascular disease, atrial fibrillation, and smoking), and common medication use after stroke (aspirin, warfarin, and atorvastatin). bBonferroni p value cutoff = 0.05/257 miRNAs = 1.9E-4. (DOCX) [file pone.0219261.s002.docx]

**SUPPORTING INFORMATION**

Whole Blood MicroRNA Expression Associated with Stroke

**S2 Table. Most significant whole blood miRNAs in association with chronic stroke by treating miRNA expression as exposure and prevalent stroke as the dependent variable.**

| **miRNA** | **Cases**  **(No.)** | **Non-Cases**  **(No.)** | **Average expression**  **(Ct)** | | **Association results^a^** | | |
| --- | --- | --- | --- | --- | --- | --- | --- |
|  |  |  | **Cases** | **Non-Cases** | **Beta** | **SE** | ***P*-value^b^** |
| miR-574-3p | 53 | 2383 | 12.72 | 10.51 | 0.17 | 0.04 | 1.0E-04 |
| miR-483-3p | 24 | 793 | 22.13 | 23.68 | -0.37 | 0.10 | 1.8E-04 |
| RNU48-b2 | 54 | 2407 | 4.25 | 3.24 | 0.19 | 0.11 | 8.3E-02 |
| miR-28-5p | 54 | 2397 | 14.55 | 12.13 | 0.23 | 0.10 | 1.8E-02 |
| miR-320b | 53 | 2417 | 11.30 | 9.22 | 0.15 | 0.08 | 5.5E-02 |
| RNU48-a2 | 54 | 2396 | 4.32 | 3.48 | 0.40 | 0.18 | 2.7E-02 |
| U6-snRNA-a1 | 51 | 2313 | 8.92 | 11.13 | -0.32 | 0.11 | 4.8E-03 |
| RNU48-b1 | 54 | 2405 | 4.24 | 3.47 | 0.19 | 0.13 | 1.5E-01 |
| miR-324-3p | 54 | 2386 | 11.69 | 10.27 | 0.22 | 0.09 | 1.2E-02 |
| miR-625-3p | 47 | 2358 | 15.15 | 15.48 | -0.17 | 0.06 | 6.1E-03 |

Abbreviations: Ct, Cycle threshold.

^a^Model adjusted for age, sex, technical factors, time interval between prior stroke and RNA extraction (time interval for non-cases is zero years), potentially confounding clinical variables (systolic blood pressure, hypertension treatment, diabetes, previous cardiovascular disease, atrial fibrillation, and smoking), and common medication use after stroke (aspirin, warfarin, and atorvastatin).

^b^Bonferroni *p* value cutoff = 0.05/257 miRNAs = 1.9E-4
