# Supplementary material for: Whole blood microRNA expression associated with stroke: Results from the Framingham Heart Study
Source: PLoS One. 2019 Aug 8;14(8):e0219261. doi: 10.1371/journal.pone.0219261 (PMC6687152; doi:10.1371/journal.pone.0219261)
Supplement: S3 Table — A total of 1063 genes were identified (P<2.8E-6). (DOCX) [file pone.0219261.s003.docx]

**SUPPORTING INFORMATION**

Whole Blood MicroRNA Expression Associated with Stroke

**S3 Table. Top genes whose expression in blood was associated with miR-574-3p.** A total of 1063 genes were identified (*P*<2.8E-6).

| **Gene** | **Beta** | **SE** | **P-value** |
| --- | --- | --- | --- |
| CSF2RB | -0.0178 | 0.0015 | 1.4E-31 |
| CCDC109B | 0.0170 | 0.0016 | 4.2E-27 |
| ARAP1 | -0.0168 | 0.0017 | 5.5E-23 |
| TLE3 | -0.0127 | 0.0013 | 1.6E-22 |
| KIAA1949 | -0.0120 | 0.0012 | 2.2E-22 |
| NOTCH1 | -0.0122 | 0.0013 | 3.7E-22 |
| DYSF | -0.0181 | 0.0019 | 5.6E-22 |
| USP34 | -0.0104 | 0.0011 | 1.2E-21 |
| BRWD3 | -0.0125 | 0.0013 | 5.1E-21 |
| KIAA0247 | -0.0125 | 0.0013 | 1.7E-20 |
| LASP1 | -0.0120 | 0.0013 | 1.8E-20 |
| LITAF | -0.0134 | 0.0015 | 2.6E-20 |
| IL17RA | -0.0133 | 0.0014 | 2.8E-20 |
| FMNL1 | -0.0125 | 0.0014 | 3.6E-20 |
| PREX1 | -0.0151 | 0.0017 | 5.5E-20 |
| RARA | -0.0101 | 0.0011 | 7.6E-20 |
| H2AFZ | -0.0208 | 0.0023 | 8.1E-20 |
| SMCHD1 | -0.0152 | 0.0017 | 1.8E-19 |
| CORO1A | -0.0127 | 0.0014 | 2.8E-19 |
| BOD1L | -0.0154 | 0.0017 | 3.3E-19 |
