# Supplementary material for: Whole blood microRNA expression associated with stroke: Results from the Framingham Heart Study
Source: PLoS One. 2019 Aug 8;14(8):e0219261. doi: 10.1371/journal.pone.0219261 (PMC6687152; doi:10.1371/journal.pone.0219261)
Supplement: S4 Table — (DOCX) [file pone.0219261.s004.docx]

**SUPPORTING INFORMATION**

Whole Blood MicroRNA Expression Associated with Stroke

**S4 Table. Enrichment of genes expressed in blood associated with miR-574-3p.**

| KEGG pathway | Number of Genes in Pathway | Ratio of enrichment | *P* value | FDR Q-value | Overlapping genes in the pathway |
| --- | --- | --- | --- | --- | --- |
| Non-alcoholic fatty liver disease (NAFLD) | 151 | 3.19 | 2.66E-09 | 8.1E-07 | *COX4I1; AKT1; AKT2; MLXIP; PIK3R5; GSK3A; GSK3B; IL6R; LEPR; MAP3K11; NDUFA3; NDUFA6; NDUFA9; NDUFAB1; NDUFB5; NDUFB8; NDUFB10; NDUFC1; NDUFC2; NDUFS3; NDUFS4; NDUFS5; NDUFV3; ADIPOR1; PIK3CA; PIK3CD; NDUFA12; BAX; RELA; RXRA; TNFRSF1A; XBP1* |
| Alzheimer's disease | 171 | 2.73 | 2.16E-07 | 3.3E-05 | *ADAM10; COX4I1; GSK3B; APAF1; APP; ITPR2; LRP1; MME; NDUFA3; NDUFA6; NDUFA9; NDUFAB1; NDUFB5; NDUFB8; NDUFB10; NDUFC1; NDUFC2; NDUFS3; NDUFS4; NDUFS5; NDUFV3; ATP2A3; ATP5C1; PLCB2; ATP5O; PPP3CA; MAPK3; NDUFA12; SNCA; TNFRSF1A; NAE1* |
| Fc gamma R-mediated phagocytosis | 93 | 3.40 | 5.00E-07 | 5.1E-05 | *WASF2; AKT1; AKT2; PIK3R5; GSN; INPP5D; INPPL1; LIMK2; LYN; MARCKS; PAK1; ASAP1; PIK3CA; PIK3CD; PRKCD; MAPK3; PTPRC; RAC2; VASP; VAV1; GAB2* |
| Proteoglycans in cancer | 202 | 2.38 | 3.09E-06 | 2.3E-04 | *CTTN; AKT1; AKT2; FLNA; PIK3R5; PDCD4; GRB2; HCLS1; HIF1A; IGF1R; ITGA5; ITGB3; ITPR2; MMP9; MSN; PPP1R12A; PAK1; PDPK1; PIK3CA; PIK3CD; PLAUR; PPP1CA; PPP1CB; MAPK3; PTPN6; PXN; ROCK1; THBS1; CBL; IQGAP1; ARHGEF1; ROCK2* |
| Platelet activation | 122 | 2.84 | 4.12E-06 | 2.5E-04 | *RASGRP2; ADCY7; AKT1; AKT2; PIK3R5; ITGA2B; ITGB3; ITPR2; LYN; PPP1R12A; PIK3CA; PIK3CD; PLCB2; PPP1CA; PPP1CB; MAPK3; PTGS1; ROCK1; TLN1; VASP; FERMT3; ARHGEF1; ROCK2* |
| Focal adhesion | 203 | 2.30 | 9.59E-06 | 4.8E-04 | *DIAPH1; AKT1; AKT2; FLNA; PIK3R5; GRB2; GSK3B; IGF1R; ITGA2B; ITGA5; ITGB3; PPP1R12A; PAK1; PDPK1; PIK3CA; PIK3CD; PPP1CA; PPP1CB; MAPK3; PXN; RAC2; BCL2; ROCK1; THBS1; TLN1; VASP; VAV1; ZYX; ACTN4; ACTN1; ROCK2* |
| Regulation of actin cytoskeleton | 216 | 2.23 | 1.31E-05 | 5.7E-04 | *WASF2; IQGAP2; DIAPH1; PIK3R5; GSN; ITGA2B; ITGA5; ITGAL; ITGAM; ITGAX; ITGB2; ITGB3; LIMK2; MSN; MYH9; PPP1R12A; PAK1; PIK3CA; PIK3CD; PPP1CA; PPP1CB; MAPK3; PXN; RAC2; ROCK1; VAV1; ACTN4; ACTN1; IQGAP1; FGD3; ARHGEF1; ROCK2* |
| B cell receptor signaling pathway | 73 | 3.30 | 1.73E-05 | 6.6E-04 | *AKT1; AKT2; PIK3R5; GRB2; GSK3B; INPP5D; INPPL1; LYN; PIK3CA; PIK3CD; PPP3CA; MAPK3; PTPN6; RAC2; RELA; VAV1* |
| Leukocyte transendothelial migration | 118 | 2.68 | 2.70E-05 | 8.2E-04 | *PIK3R5; ITGAL; ITGAM; ITGB2; MMP9; MSN; NCF2; F11R; PECAM1; PIK3CA; PIK3CD; PXN; RAC2; ROCK1; SIPA1; VASP; VAV1; CXCR4; ACTN4; ACTN1; ROCK2* |
| Thyroid hormone signaling pathway | 118 | 2.68 | 2.70E-05 | 8.2E-04 | *NCOA2; CREBBP; AKT1; AKT2; MED13L; PIK3R5; GSK3B; HIF1A; ITGB3; NOTCH1; NOTCH2; PDPK1; PIK3CA; PIK3CD; PLCB2; MAPK3; RXRA; TSC2; NCOA1; MED12; MED13* |
