# Supplementary material for: Whole blood microRNA expression associated with stroke: Results from the Framingham Heart Study
Source: PLoS One. 2019 Aug 8;14(8):e0219261. doi: 10.1371/journal.pone.0219261 (PMC6687152; doi:10.1371/journal.pone.0219261)
Supplement: S5 Table — Two genes were significant after Bonferroni correction (P<8.9E-7). (DOCX) [file pone.0219261.s005.docx]

**SUPPORTING INFORMATION**

Whole Blood MicroRNA Expression Associated with Stroke

**S5 Table. Top genes whose expression was associated with miR-574-3p in brain samples collected from the ROS/MAP project.** Two genes were significant after Bonferroni correction (*P*<8.9E-7).

| Gene | Beta | SE | P-value |
| --- | --- | --- | --- |
| *DBNDD2* | **17.64** | **3.24** | **7.7E-08** |
| *ELOVL1* | **3.87** | **0.75** | **3.3E-07** |
| *DUSP10* | 0.31 | 0.06 | 9.3E-07 |
| *LIPE* | 0.79 | 0.17 | 2.3E-06 |
| *KLK6* | 2.75 | 0.58 | 2.6E-06 |
| *DNAH17* | 0.41 | 0.09 | 4.3E-06 |
| *TF* | 23.18 | 5.13 | 7.6E-06 |
| *RHOG* | 2.76 | 0.62 | 1.1E-05 |
| *THEMIS2* | 0.87 | 0.19 | 1.1E-05 |
| *LHPP* | 6.39 | 1.45 | 1.2E-05 |
| *EVI2A* | 2.37 | 0.54 | 1.3E-05 |
| *TMEM63A* | 3.66 | 0.83 | 1.4E-05 |
| *GJB1* | 2.05 | 0.47 | 1.6E-05 |
| *FA2H* | 1.99 | 0.46 | 1.8E-05 |
| *RNASE1* | 11.60 | 2.71 | 2.3E-05 |
| *LDB3* | 0.77 | 0.18 | 2.5E-05 |
| *SLC47A1* | 0.36 | 0.08 | 2.8E-05 |
| *MLPH* | 0.11 | 0.03 | 2.8E-05 |
| *RP11-288G11.3* | 1.63 | 0.39 | 3.4E-05 |
| *GPR62* | 0.60 | 0.14 | 3.4E-05 |
